# Effect of silver diamine fluoride solution application on the bond strength of dentine to adhesives and to glass ionomer cements: a systematic review

**DOI:** 10.1186/s12903-020-1030-z

**Published:** 2020-02-05

**Authors:** Meng Jiang, May Lei Mei, May Chun Mei Wong, Chun Hung Chu, Edward Chin Man Lo

**Affiliations:** 1 Faculty of Dentistry, The University of Hong Kong, 34 Hospital Road, Sai Ying Pun, Hong Kong, China; 2 Faculty of Dentistry, The University of Otago, 310 Great King Street, Dunedin, 9016 New Zealand; 3 Dental Public Health, The Prince Philip Dental Hospital, 3F, 34 Hospital Road, Sai Ying Pun, Hong Kong, China

**Keywords:** Silver diamine fluoride, Bond strength, Dentine, Adhesives, Glass ionomer cement, Systematic review

## Abstract

**Background:**

Silver diamine fluoride (SDF) solution gains increasing popularity in arresting dentine caries in clinical practice. The aim of this systematic review was to summarize the findings from laboratory studies on the influence of SDF application on the bond strength of dentine to various adhesives and to glass ionomer cements (GICs).

**Methods:**

Two independent reviewers conducted a literature search in the databases Medline, Ovid, PubMed and Web of Science until 15th August 2019 using the search keywords [‘bond strength’] AND [‘silver diamine fluoride’ OR ‘silver diammine fluoride’ OR ‘SDF’ OR ‘silver fluoride’ OR ‘diamine silver fluoride’]. Articles investigating the effect of SDF application on the bond strength of dentine to various adhesives and to GICs were included in this review. Information on how SDF application influenced the bond strength was extracted from the included articles. Besides, related information, e.g. test method of bond strength, concentration and brand of SDF, type of adhesive system and GIC, testing dental substrate, protocol of specimen preparation, and failure mode was also reviewed.

**Results:**

A total of 13 articles were included in this review, with 8 and 6 studies investigating the effect of SDF application on the bond strength of dentine to various adhesives and to GICs, respectively. Sound dentine as well as demineralized dentine created by chemical methods, e.g. immersing in a demineralizing solution, was commonly adopted as the testing dental substrate. The microtensile bond strength (mTBS) test was the predominant method employed. However, the bond strength values had large variations among studies, ranging from <10 to 162 Mpa. Regarding the bond strength to different adhesives, 4 studies indicated that SDF application followed by rinsing with water had no significant influence. However, another 4 studies reported reduced bond strength after SDF application. Regarding the bond strength to GICs, 4 studies concluded that SDF application had no adverse impact on the bond strength.

**Conclusions:**

No solid conclusion can be drawn on the effect of SDF application on the bond strength of dentine to adhesives and to GICs due to the high degree of variation of the included studies.

## Background

Silver diamine fluoride (SDF), containing diamine-silver ion and fluoride ion, is a colorless alkaline solution. Diamine-silver ion is a complex with two ammonia molecules attached to a silver ion, which makes it more stable and less oxidizing than silver ion [1]. The combination of silver and fluoride in an alkaline solution has a synergistic effect in arresting dentine caries, which makes SDF different from other fluoride agents [2]. SDF can inhibit demineralization and preserve the collagen in dentine from degradation [3]. There is a significant increase in microhardness with an elevated level of calcium and phosphorus in the outmost surface layer of the SDF arrested dentine caries lesion [4, 5]. Additionally, SDF can react with calcium and phosphate ions to produce fluorohydroxyapatite with reduced solubility, which is considered as one of the main factors in arresting caries lesions [6].

With a better understanding of pathology, dental caries is now known as a biofilm-mediated, sugar-driven, multifactorial dynamic disease [7]. This dynamic process involves alternating periods of demineralization and remineralization of dental hard tissues. The perspective that demineralized dental tissues have potential to remineralize is increasingly accepted by dental professionals. Instead of performing extended cavity preparation when treating a carious lesion, it is recommended to use a minimally invasive approach to preserve not only sound tooth tissue but also tissues with the potential to remineralize [8, 9]. Indeed, non-invasive methods with no removal of carious tissues are accepted options for dental caries management [10].

Topical application of SDF, a non-invasive treatment for caries, has been shown to be effective in arresting dentine caries in young children [11–14] as well as in older adults [15]. The application of SDF solution is easy, low-cost and painless. There is no need to remove carious dental tissues before application of SDF [16], which simplifies the treatment procedure and reduces the discomfort of patient. Although SDF application can arrest active caries and prevent development of dental complications, there are limitations of what SDF treatment can achieve. For example, the black stain on the SDF-arrested caries lesions may cause esthetic concerns, and the chewing function of the cavitated teeth may not be improved because the tooth cavities are not filled up. Placing a dental restoration may be a solution to these problems. Tooth-coloured restorative materials can be used to restore the caries cavity to cover the black stain caused by SDF, and to reshape the tooth contour to improve chewing ability and dental appearance. It was reported that placement of GIC restorations in SDF-treated caries lesions with atraumatic restorative treatment (ART) approach can improve parental satisfaction with their child’s teeth [17]. Hence, it seems promising to incorporate SDF application into caries management together with restorative treatment.

The development of adhesive technology has revolutionized restorative dentistry. Dental adhesive systems are commercially categorized into generations reflecting the handling technique or advances in formulations [18]. For dental adhesives bonding to dentine, there are two main approaches. One is to completely remove the smear layer and superficial demineralized tissues by a strong acid, and the other is to partially dissolve and incorporate the smear layer into the adhesive interface by a mild or intermediate acid. Therefore, two main categories of adhesives, known as ‘etch-and-rinse’ and ‘self-etch’ systems, are commercially available [19, 20]. In the etch-and-rinse system, before application of primer and adhesive, an acid is used to etch the dental substrate and then followed by rinsing with water. In contrast, in the self-etch system, the acid etching and rinsing with water step is omitted, and a mild or intermediate acid is used to partially dissolve and modify the smear layer. In both systems, adhesion is achieved by micromechanical retention to the underlying etched dental substrates. An additional chemical bonding between dental substrates and adhesives can be observed in the self-etch system [18].

In addition, glass ionomer cement (GIC), an acid-based material, is commonly used to restore dental cavities, especially in the ART approach. By mixing the GIC powder and liquid, an acid-based chemical reaction occurs. When the acid attacks the glass powder, metal ions (Ca^2+^ and Al^3+^) are released to form crosslinks with the polyalkenoic acid chains [21]. The hardness of GIC materials is the combined effect of crosslinking of metal ions and neutralization of the polyalkenoate molecules. Adhesion of GIC to dentine is achieved by both chemical bonding and micromechanical interlocking [22]. GIC may be considered as a self-etching system, an effect that arises from the presence of an acid component within it. Hence, if micromechanical interlocking is enhanced, the bond strength will be increased. Other than the traditional chemical-cure system, resin-modified GIC (RMGIC) contains a polymerizable monomer 2-hydroxyethyl methacrylate (HEMA) as an additional component to achieve better adhesion [22].

As there is an increasing use of SDF for management of caries, the dentine surface of cavities may have been treated by SDF before placement of restoration. Thus, it is important to investigate whether this will affect the bonding of adhesives to dentine. The aim of this systematic review was to summarize the findings from laboratory studies on the influence of SDF application on the bond strength of dentine to different adhesives, including both etch-and-rinse and self-etch systems, and to GICs.

## Methods

This systematic review was conducted according to PRISMA guideline. The literature search was conducted by two independent reviewers to identify articles in the databases Medline, Ovid, PubMed and Web of Science until 15th August 2019, without initial time limit and language restriction. The search keywords were [‘bond strength’] AND [‘silver diamine fluoride’ OR ‘silver diammine fluoride’ OR ‘SDF’ OR ‘silver fluoride’ OR ‘diamine silver fluoride’]. The identified articles from the four databases were checked for duplication. After removing the duplicates, titles and abstracts of the potential eligible articles were screened by the same two independent reviewers. The inclusion criterion was laboratory study which reported on the effect of SDF or sliver fluoride application on the bond strength of dentine to various adhesives and/or to GICs. Articles were excluded if (1) not related to bond strength test, and (2) the testing dental substrate was enamel only. Afterwards, full texts of the remaining articles were retrieved and article which did not report bond strength values were excluded. The following data were extracted from the included studies: test method of bond strength, value of bond strength, concentration and brand of SDF, type of adhesive system and GIC, testing dental substrate, specimen preparation method and failure mode. The same two independent reviewers conducted data extraction. If there was disagreement on the inclusion of a study or extraction of data, discussion with a third independent investigator was held so as to arrive at a consensus.

## Results

**Fig. 1:**
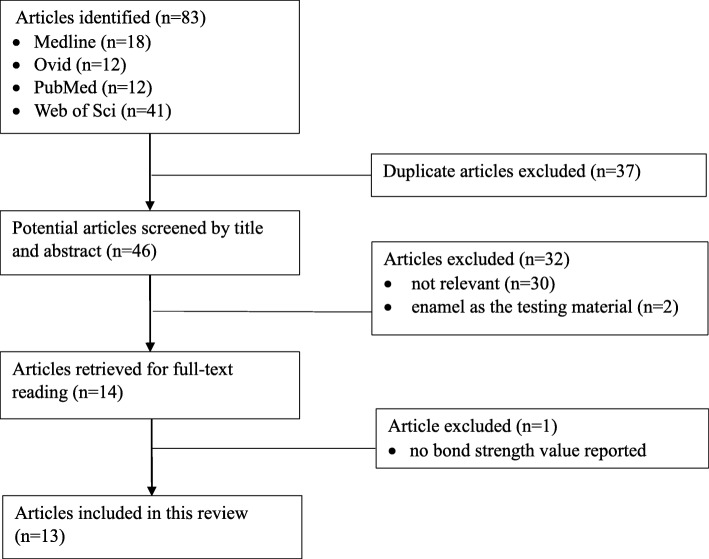
Flowchart of study selection process

A total of 83 articles were identified from the selected databases and 37 duplicated articles were removed (Fig. 1). After screening the title and abstract, 32 articles were excluded for not related to bond strength test (*n* = 30), and for using enamel only as the testing dental substrate (*n* = 2). Fourteen articles were retrieved for full-text reading. One article was excluded because no bond strength value was reported. Finally, 13 articles were included in this review, with 8 and 6 studies investigating the effect of SDF application on the bond strength of dentine to various adhesive systems and to GICs, respectively.

Table 1 presents the main characteristics of the included studies. Various bond strength test methods were used in the included studies, including tensile bond strength (TBS), shear bond strength (SBS), microtensile bond strength (mTBS), and microshear bond strength (mSBS) tests. The predominant test adopted was mTBS test, which was used in seven studies [23, 26, 28, 29, 31–33]. The SBS and mSBS test was used in three [24, 25, 34] and two [27, 30] studies, respectively. Regarding the dental substrate, human tooth dentine was used in all of the studies, except one study conducted two decades ago which used bovine dentine [35]. Sound dentine of human teeth was the most commonly used dental substrate [25, 27–34], while demineralized dentine created by immersing sound dentine in demineralized solution was also adopted in some studies [23, 24, 29, 31]. Only one study used natural carious dentine from extracted primary molars [26]. In the majority of the studies, 38% SDF solution was used. Apart from that, a commercial SDF/KI product (Riva Star, SDI Limited, Victoria, Australia) was adopted in four studies [24, 25, 30, 32]. Besides, it was found that two included studies used a saturated potassium iodine (KI) solution instead of commercially available products. Even using the same material, the SDF application protocols were quite different among the included studies. For example, in several studies, SDF was applied and left to air-dry before the bonding process, whereas in other studies, application of SDF was followed by rinsing with water.

**Table 1:** Main characters of the included studies

Authors (Year)	Method	SDF	Teeth	Dental substrate	Adhesives	Protocol^f^	Other intervention
Van Duker et al., [23] (2019)	mTBS	38% SDF^a^ 38% SDF^a^/KI^b^	Posterior teeth	D-dentine	Etch-and-rinse system (Scotchbond Universal Adhesive, 3 M, St. Paul, MN, USA)	Rinse	
Zhao et al., [24] (2019)	SBS	38% SDF^c^ 35–40% SDF/KI^d^	Third molar	D-dentine	GIC (Ketac-Molar, 3 M/ESPE Dental Products, St, Paul, MN, USA)	Air-dry/Rinse	
Gupta et al., [25] (2019)	SBS	35–40% SDF/KI^d^	Molar	S-dentine	RMGIC (GC Gold Label 2 LC, GC Co., Japan)	Rinse	
Puwanawiroj et al., [26] (2018)	mTBS	38% SDF^c^	Primary molar	Natural carious dentine	GIC (Fuji IX GP Extra Capsule, GC America Inc.)	Rinse + Polish	
Lutgen et al., [27] (2018)	mSBS	38% SDF^a^	Posterior teeth	S-dentine	Self-etch system (Scotchbond Universal Adhesive, 3 M, St. Paul, MN, USA; Clearfil SE Bond 2, Kuraray Noritake, Tokyo, Japan) Etch-and-rinse system (Scotchbond Universal Adhesive, 3 M, St. Paul, MN, USA; Scotchbond Etchant, 3 M, St. Paul, MN, USA)	Air-dry/Rinse/Polish	
Wu et al., [28] (2016)	mTBS	38% SDF^c^	Primary molar	S-dentine	Etch-and-rinse system (Prime & Bond NT, DENTSPLY Caulk, Milford, Del., USA)	Rinse	
Wang et al., [29] (2016)	mTBS	38% SDF^c^	Molar	S-dentine D-dentine	GIC (Fuji IX, GC Co., Tokyo, Japan)	Air-dry	Light illumination
Selvaraj et al., [30] (2016)	mSBS	35–40% SDF/KI^d^	Third molar	S-dentine	Etch-and-rinse system (Adper Single Bond 2, 3 M ESPE, St Paul, MN, USA); Self-etch system (Adper Easy One, 3 M ESPE, St Paul, MN, USA)	Rinse	
Kucukyilmaz et al., [31] (2016)	mTBS	38% SDF^c^	Molar	S-dentine D-dentine	Self-etch system (Clearfil SE Bond, Kuraray, Tokyo, Japan)	Not reported	Laser
Koizumi et al., [32] (2016)	mTBS	35–40% SDF/KI^d^	Permanent molar	S-dentine	Etch-and-rinse system (Optibond FL; Kerr, USA) 2-step self-etching systems (Clearfil Liner Bond F; Kuraray Noritake Dental, Japan; and Optibond Versa; Kerr, USA) RMGIC (Riva Bond LC; SDI Australia)	Not reported	
Quock et al., [33] (2012)	mTBS	38% SDF^c^ 12% SDF^e^	Molar	S-dentine	Self-etch system (Peak SE, Ultradent, South Jordan, UT, USA) Etch-and-rinse system (Peak LC, Ultradent, South Jordan, UT, USA)	Rinse	
Knight et al., [34] (2006)	SBS	1.8 M AgF solution saturated KI solution	Third molar	S-dentine	GIC (Fuji VII, GC Co., Japan)	Rinse/Air-dry	
Soeno et al., [35] (2001)	TBS	38% SDF^c^	Bovine anterior teeth	Bovine dentine	2 luting systems (Super-Bond C & B, Sun Medical Co. Ltd., Moriyama, Japan; and Panavia Fluoro Cement, Kuraray Co. Ltd., Osaka, Japan)	Air-dry + Rinse	

*mTBS* Microtensile bond strength, *mSBS* Microshear bond strength, *TBS* Tensile bond strength, *SBS* Shear bond strength, *SDF* Silver diamine fluoride, *KI* Potassium iodine, *D-dentine* Demineralized dentine, *S-dentine* Sound dentine, *RMGIC* Resin-modified glass ionomer cement

^a^38% SDF solution (Advantage Arrest, Elevate Oral Care, West Palm Beach, FL, USA);

^b^a saturated solution of KI (Sigma-Aldrich, St. Louis, MO, USA);

^c^38% SDF solution (Saforide; Osaka, Japan);

^d^SDF/KI product (Riva Star, SDI Limited, Victoria, Australia), consisting of 35–40% silver fluoride and a saturated solution of KI;

^e^12% SDF solution (Ancarie 12% Cariostatico, Maquira Dental Products, Maringa, PR, Brazil); Protocol^f^, procedure performed after application of SDF solution

Regarding the bond strength of dentine to ‘etch-and-rinse’ and ‘self-etch’ adhesives, inconsistent findings were reported (Table 2). Four studies found that SDF application followed by rinsing with water immediately had no significant influence on the bond strength [23, 28, 30, 33]. In contrast, two studies concluded that after the above-mentioned procedure, the bond strength of dentine to adhesives was reduced significantly [27, 35]. Another two studies drew a similar conclusion that the bond strength was jeopardized after SDF application, but they did not clearly describe the procedure taken after SDF application [31, 32].

**Table 2:** Main findings of the effect of SDF application on the bond strength of dentine to etch-and-rinse and self-etch adhesive systems

Authors (Year)	Sample size per group	Mean bond strength value (Mpa)			*Analytical tool (magnification)* Dominant failure mode	Main findings	Summary
		Control group	Test group				
			SDF	SDF/KI			
Van Duker et al., [23] (2019)	10	Etch-and-rinse system			*Not reported*	• Application of SDF followed by rinsing with water had no significant influence on the mTBS of demineralized dentine to the adhesive • Application of SDF/KI followed by rinsing with water reduced the mTBS of demineralized dentine to the adhesive	ns/decrease
		23.5 ± 10.7	19.8 ± 8.4	7.9 ± 6.6*			
Lutgen et al., [27] (2018)	10	Self-etch system (Scotchbond Universal Adhesive)			*Light microscope* *(5x)* (1) Control: Mixed (2) SDF (air-dry): Adhesive (3) SDF (rinse): Adhesive (4) SDF (polish): Adhesive (Scotchbond Universal Adhesive) Mixed (Scotchbond Etchant and Clearfil SE Bond 2)	• Application of SDF followed by either air-drying or rinsing with water reduced the mSBS of sound dentine to the three study adhesives • Application of SDF followed by polishing reduced the mSBS of sound dentine to the Scotchbond Universal Adhesive, whereas there were no significant influences on the other two adhesive systems.	decrease
		38.4 ± 4.3	12.8 ± 1.7* (air-dry) 19.9 ± 9.4* (rinse) 28.7 ± 7.1* (polish)				
		Etch-and-rinse system (Scotchbond Universal Adhesive + Scotchbond Etchant)					
		44.5 ± 4.6	24.0 ± 2.7* (air-dry) 31.9 ± 8.2* (rinse) 44.3 ± 4.6 (polish)				
		Self-etch system (Clearfil SE Bond 2)					
		40.0 ± 2.3	12.9 ± 3.7* (air-dry) 28.6 ± 3.8* (rinse) 35.7 ± 4.7 (polish)				
Wu et al., [28] (2016)	12	Etch-and-rinse system			*Visual inspection and SEM* Adhesive	• Application of SDF followed by rinsing with water had no significant influence on the mTBS of sound dentine to the adhesive	ns
		162.09 ± 81.08	139.85 ± 88.53				
Selvaraj et al., [30] (2016)	15	Etch-and rinse system (Adper Single Bond 2)			*SEM* Adhesive	• Application of SDF/KI followed by rinsing with water had no significant influence on the mSBS of sound dentine to the two adhesives	ns
		28.69 ± 1.49		29.52 ± 1.13			
		Self-etch system (Adper Easy One)					
		28.47 ± 0.53		29.04 ± 0.77			
Kucukyilmaz et al., [31] (2016)	32	S-dentine 36.79 ± 5.37 D-dentine 30.10 ± 5.88 S-dentine+laser 37.06 ± 5.53 D-dentine+laser 34.78 ± 5.34	31.87 ± 7.54* 18.89 ± 2.28* 22.50 ± 6.49* 17.65 ± 3.15*		*Light microscope* *(40x)* Adhesive	• Application of SDF reduced the mTBS of both sound and demineralized dentine to adhesives • Laser irradiation adversely affected the mTBS of SDF-treated dentine	decrease
Koizumi et al., [32] (2016)	10	Etch-and-rinse system (Optibond FL)			*SEM* Control: Cohesive SDF/KI: Mixed	• Application of SDF/KI reduced the mTBS of sound dentine to the three adhesives	decrease
		32.1 ± 1.2		21.4 ± 9.4*			
		Self-etch system (Optibond Versa)					
		35.0 ± 3.9		9.6 ± 2.0*			
		Self-etch system (Clearfil Liner Bond F)					
		28.4 ± 8.4		10.8 ± 2.1*			
Quock et al., [33] (2012)	7	Self-etch system (Peak SE)			*Not reported* Adhesive and/or cohesive	• Application of SDF followed by rinsing with water had no significant influence on the mTBS of sound dentine to the two adhesives	ns
		32.96 ± 6.72	23.99 ± 8.04 (12% SDF) 32.73 ± 10.54 (38% SDF)				
		Etch-and-rinse system (Peak LC)					
		30.81 ± 2.55	39.00 ± 11.46 (12% SDF) 34.41 ± 10.48 (38% SDF)				
Soeno et al., [35] (2001)	5	Super-bond C&B			*SEM* Super-bond C&B Control: Cohesive SDF: Adhesive Panavia Fluoro Cement Control: Mixed SDF: Adhesive	• Application of SDF reduced the TBS of bovine dentine to the two adhesives	decrease
		10.2 ± 1.9	6.0 ± 2.2*				
		Panavia Fluoro Cement					
		7.1 ± 1.2	2.6 ± 1.1*				

*p < 0.05, compared with control group; *SEM* Scanning electron microscope, *S-dentine* Sound dentine, *D-dentine* Demineralized dentine, *ns* No significant difference

Table 3 shows the findings regarding the bond strength of dentine to GICs. Three studies made a conclusion that application of SDF had no adverse influence on the bond strength [24, 26, 29]. In addition, one study showed that SDF application followed by light illumination of dentine surface resulted in a higher bond strength value (*p* < 0.05) [29]. Another study also reported a higher bond strength value after SDF application (*p* < 0.05) [25]. On the contrary, a study found that leaving the applied SDF to air-dry on the dentine surface resulted in a lower bond strength value (p < 0.05), while SDF application followed by rinsing with water immediately had no statistically significant impact on bond strength [34].

**Table 3:** Main findings of effect of SDF application on the bond strength of dentine to GIC/RMGIC

Authors (Year)	Sample size per group	Bond strength (Mpa)			*Analytical tool (magnification)* Dominant failure mode	Main findings	Summary
		Control group	Test group				
			SDF	SDF/KI			
Zhao et al., [24] (2019)	19	2.6 ± 1.1	2.3 ± 0.9	3.0 ± 1.4	*Light microscope* *(20x)* Adhesive	• Application of SDF followed by air-drying had no significant influence on the SBS of demineralized dentine to GIC • Application of SDF/KI followed by rinsing with water had no significant influence on the SBS of demineralized dentine to GIC	ns
Gupta et al., [25] (2019)	8	10.5 ± 10.5 (water) 8.9 ± 4.6 (polyacrylic acid) 9.7 ± 5.3 (chlorhexidine)		22.3 ± 4.4*	*Not reported*	• Compared to polyacrylic acid, chlorhexidine and water, application of SDF/KI followed by rinsing with water increased the SBS of sound dentine to RMGIC	increase
Puwanawiroj et al., [26] (2018)	40	6.3 ± 4.6	7.4 ± 5.1		*Light microscope* *(40x)* Mixed	• Application of SDF followed by rinsing with water and polishing had no significant influence on the mTBS of carious dentine to GIC	ns
Wang et al., [29] (2016)	20	24 h S-dentine Water 4.00 ± 1.68 D-dentine Water 3.84 ± 1.47 7 days S-dentine Water 5.83 ± 2.04 D-dentine 6.04 ± 2.24	4.25 ± 1.80 4.80 ± 2.53 (light) 4.74 ± 1.41 6.25 ± 2.60* (light) 5.19 ± 1.77 4.53 ± 2.34 (light) 6.21 ± 3.28 6.69 ± 2.72 (light)		*Light microscope* *(40x)* Cohesive	• Compared to no treatment and SDF treatment only, combination of SDF application and light illumination increased the mTBS of demineralized dentine to GIC 24 h after bonding, but this effect was not found after 7 days • Application of SDF with/without light illumination had no significant influence on the mTBS of sound dentine to GIC	increase/ns
Koizumi et al., [32] (2016)	10	18.4 ± 5.6		14.5 ± 5.2*	*SEM* Adhesive	• Application of SDF/KI reduced the mTBS of sound dentine to RMGIC	decrease
Knight et al., [34] (2006)	10	2.40 ± 0.88		2.83 ± 1.39 (rinse) 1.53 ± 0.74* (air-dry)	*Not reported*	• Application of SDF/KI and leaving the precipitate on the dentine surface reduced the SBS of sound dentine to GIC • Application of SDF/KI followed by rinsing with water to remove precipitate on the surface had no significant influence on the SBS of sound dentine to GIC	decrease/ns

*p < 0.05, compared with control group, *SEM* Scanning electron microscope, *S-dentine* Sound dentine, *D-dentine* Demineralized dentine, *ns* No significant difference

In the included studies, failure mode of the interface was examined by a light microscope and/or a scanning electronic microscope (SEM). Failure modes were classified into three main types, (1) adhesive failure at the interface; (2) cohesive failure within either the material or dentine; (3) mixed failure with combination of both. It was found that the dominant failure mode varied among the included studies. In general, in the studies that used various adhesive systems, adhesive failure was more likely to be observed in the specimens with SDF application, while more cohesive and mixed failure modes were found in the control group specimens. As for specimens bonded to GICs, two studies found that adhesive failure was the predominant mode, while cohesive and mixed failure modes were reported to occur more frequently in another two studies.

## Discussion

Inconsistent results regarding the effect of SDF application on the bond strength of dentine to adhesives and to GICs were reported in the studies included in this review. Lack of a standard way to prepare specimen, including the SDF application protocol, is a probable reason to explain the inconsistency [36]. In some studies, after application of SDF, the dentine surface was rinsed with water immediately, while in other studies the SDF was left to naturally air-dry. Neither ways to apply SDF are close to the real clinical situation. In clinical practice, SDF is usually applied on the dentine surface without rinsing with water immediately. The patient is simply instructed not to eat or drink for half an hour after SDF application [13]. It is not practicable to have the SDF air-dried on the dentine surface after application in patients because the oral cavity is always moist with presence of saliva. Besides, several included studies polished the SDF-treated dentine surface with a 600-grit silicon carbide paper prior to the bonding procedure. This gives rise to a concern that the SDF-treated surface including the precipitates from the SDF reaction, e.g. fluorohydroxyapatite, may be removed in the polishing process. If so, the study findings could not represent the true effect of SDF application on the bond strength of dentine to adhesives. Therefore, in the preparation of specimen, it is suggested to adopt the same SDF application procedure as used in clinical practice to make the study findings more relevant and useful to dentists.

A common outcome of SDF application is the black stain on the arrested caries lesions [37]. It was proposed to apply a saturated KI solution immediately after application of SDF to minimize the black stain [34]. The iodide ions in the KI solution can react with silver ions to form silver iodide (AgI) which appears as a yellowish precipitate. Four of the included studies used a commercial product which consists of two capsules, one containing SDF solution and the other containing KI solution. These studies reported that they followed the manufacturer’s instruction, which was to apply KI on the dentine surface immediately after SDF application. A self-prepared saturated KI solution was used in two other included studies. It was speculated that the bond strength was not adversely affected as long as the precipitates of SDF/KI reaction on the dentine surface was rinsed away after the application, otherwise leaving the SDF/KI precipitates on the dentine surface reduced the bond strength significantly [34]. Three included studies in which the precipitates of SDF/KI reaction on the dentine surface were rinsed away with water found no adverse influence on the bond strength [24, 25, 30]. On the contrary, one study reported that with a rinsing procedure after SDF/KI application the bond strength still reduced significantly [23]. Another study also found that bond strength of dentine to various adhesive systems decreased after SDF/KI application [32], but it did not report whether rinsing with water was carried out after the application. The report simply mentioned that the SDF/KI application procedure followed the manufacturer’s instructions, in which we found no recommendation on rinsing with water [38]. Hence, according to the findings of the included studies, we cannot draw a conclusion on the influence of SDF/KI application on the bond strength of dentine to adhesives irrespective of rinsing off the precipitates on the dentine surface or not.

The reported failure modes of interfaces varied greatly among the included studies in this review. It should be cautious when interpreting the results because these studies employed different microscopic analytical tools. A light microscope, under magnification ranging from 5x to 40x, was used to examine the interface of specimens in some of the included studies, while SEM or naked eye visual inspection was used in other studies. The decision based solely on light microscope or naked eye visual inspection may not be accurate. A study reported that cohesive failure interface determined by a light microscope showed exposed dentinal tubules on the surface when examined under SEM [39]. Thus, it is suggested that SEM at high magnification should be used for proper determination of failure mode [40]. Regarding cohesive failure mode, rather than indicating a strong interface bonding, it may reflect a mixture of mechanical properties of the different materials involved [40]. Cohesive failure may be caused by several reasons, for example, errors in alignment of the specimen along the long axis of the testing device [41], microcracks of the specimen produced during cutting or trimming [42], and the brittleness of the material involved [43]. It is recommended to discard cohesive failure specimens and only data from specimens with adhesive failure or mixed failure with small region (< 10%) involved should be selected for bond strength calculation [40]. However, none of the included studies excluded cohesive failure specimens from their bond strength analysis, which may be another reason for the large variations in bond strength values and the inconsistent results among the included studies.

Two decades ago, shear and tensile bond strength tests were performed exclusively in specimens with a relatively large bonded surface, usually 3 to 6 mm in diameter (approximately 7 to 28 mm^2^) [40]. However, the validity of these test results was questioned due to the heterogeneity of the stress distribution at bonded interface. It is suggested that a very small surface has a better stress distribution so that more adhesive failures can be generated. Thus, specimen with small bonding region (i.e. below 2mm^2^) is adopted in the mTBS and mSBS tests which have gained increasing popularity in the recent 20 years [43]. Compared to the traditional TBS test with a relatively large bonded surface, the mTBS test has several advantages, such as proportionally more adhesive failures generated, possibility of measuring a relatively high bond strength value and more specimens can be harvested from one tooth [43]. In addition, the mTBS test is found to have a larger discriminative power than the SBS test [36]. The SBS test is considered to have very little value in the prediction of clinical performance, whereas the mTBS value was reported to be associated with the retention rate of Class V restorations in clinical studies [44, 45]. However, a study reported that there was no correlation between bond strength test results and the retention rate of restorations, but only a moderate correlation between the mTBS test results and marginal discoloration of restorations [46]. Thus, further studies are needed to investigate the correlation between laboratory bond strength tests results and clinical parameters.

It should be pointed out that there are some limitations of this systematic review. In this review, quality assessment of the included studies was not performed. For systematic reviews on randomized controlled clinical trials, the Cochrane criteria is commonly adopted to assess the risk of bias of included studies arising from different aspects such as random sequence generation, group allocation concealment and blinding [47]. However, reports of laboratory studies seldom include the necessary information for quality assessment. The included studies in this review only mentioned that the specimens were randomly allocated into the test and control groups, while none of them described details about the random sequence generation and allocation concealment. Besides, no information was given on the blinding of the specimen preparation and outcome assessment. Due to the lack of information, risk of bias of the included studies could not be assessed and remained unclear.

In this review, study with relatively small sample size such as having 5 specimens in each study group was not excluded. This is because there is no consensus on the minimum sample size in laboratory studies. In fact, none of the included studies in this review reported sample size calculation. We prefer to include and report on all the relevant studies so that readers can obtain sufficient information and make their own judgements.

Meta-analysis was not conducted in the present review due to great variations in the study designs of the included studies, such as different bond strength test methods and specimen preparation protocols, including SDF application procedures. These are key factors which can influence bond strength test results. As the experimental parameters in the included studies considerably influenced the bond strength values, it was decided not appropriate to conduct inter-study comparisons in this review [36]. Instead, we only reported the bond strength values of every study group in each study to show the intra-study comparison regarding to what extent SDF application affected the bond strength values.

## Conclusions

No solid conclusion can be drawn on the effect of SDF application on the bond strength of dentine to adhesives and to GICs due to the high degree of variation of the included studies.

## Data Availability

All data generated and analyzed in this review are included within the article.

## Abbreviations

AgF: Silver fluoride
AgI: Silver iodide
ART: Atraumatic restorative treatment
GIC: Glass ionomer cement
HEMA: 2-hydroxyethyl methacrylate
KI: Potassium iodine
mSBS: microshear bond strength
mTBS: microtensile bond strength
RMGIC: Resin-modified glass ionomer cement
SBS: Shear bond strength
SDF: Silver diamine fluoride
SEM: Scanning electronic microscope
TBS: Tensile bond strength
